# Effect of Electronic Cigarettes on the Gastrointestinal System

**DOI:** 10.7759/cureus.27210

**Published:** 2022-07-24

**Authors:** Madhurima Debnath, Dipanjan Debnath, Pratiksha Singh, Yijin Wert, Vinod Nookala

**Affiliations:** 1 Internal Medicine, MedStar Franklin Square Hospital, Baltimore, USA; 2 Internal Medicine, MedStar Washington Hospital Center, Washington DC, USA; 3 Internal Medicine, Community Medical Center, Toms River, USA; 4 Internal Medicine, University of Pittsburgh Medical Center (UPMC) Harrisburg, Harrisburg, USA

**Keywords:** electronic nicotine delivery systems (ends), electronic cigarette, vomiting, diarrhea, gastrointestinal, adverse effect, vaping, smoking, nhanes, cessation

## Abstract

Introduction: In the United States, the use of electronic cigarettes (EC) has seen an exponential rise with 12.6% of adults using an EC at least once in their lifetime, with use differing by age, sex, and race. The level of nicotine exposure of EC is highly variable with its liquids containing 14.8-87.2 mg/ml of nicotine. Its use has been documented to be associated with adverse effects on the respiratory, nervous, cardiovascular, and gastrointestinal (GI) systems. Common adverse effects on the GI system include xerostomia, oral mucositis, tongue discoloration, gingivitis, gum bleeding, nausea, vomiting, gastric burning, and altered bowel habits.

Methods: A retrospective review of the National Health and Nutrition Examination Survey (NHANES) data from 2015-16 was conducted. Data regarding the use of EC and a history of vomiting and diarrhea over a period of 30 days was analyzed using SAS 9.4 (2013; SAS Institute Inc., Cary, North Carolin, United States). Additionally, data regarding age, gender, and income were also analyzed. A p-value of <0.05 was considered statistically significant. Continuous variables were analyzed using the two-sample t-test, and categorical variables were analyzed using the Chi-Square test.

Results: A total of 944 participants were included in the study. Of these, 261 participants (males 62.84%) used EC at least one day and 683 participants (males 54.76%) never used EC, during the preceding 30 days. Amongst EC users (n=261), 10.73% had a stomach or intestinal illness manifesting as vomiting or diarrhea that started during those 30 days compared to 8.64% who never used EC during the same period (n=683). However, the results did not reach statistical significance (p = 0.3208)

Conclusion: Conflicting views exist regarding the effects of EC on the GI tract. Our study demonstrated an association between EC consumption and vomiting and diarrhea. The study results are to be viewed by taking into consideration the limitations of a smaller sample, shorter duration, comorbidities, and undefined nicotine content in the EC. Although recent studies have shown no effect of EC on the oral or gut microbiota, our study findings could be attributed to EC-induced alteration of motility or irritation of the GI tract. However, further studies are required to establish a causal relationship and enunciate the mechanism by which EC components affect the GI tract. Careful consideration and diligence about the health effects of ECs are required before it is assumed to be safe as a cigarette substitute or as a means of smoking cessation.

## Introduction

Electronic cigarettes (EC) are a type of electronic nicotine delivery systems (ENDS) incorporating an “e-liquid” that forms an aerosol on heating, which can be inhaled. It contains nicotine, and varying compositions of propylene glycol, vegetable glycerin, flavorings, and other ingredients [1]. The level of nicotine exposure with EC use is also highly variable, with its liquids containing an amount of nicotine ranging from 14.8 to 87.2 mg/ml of nicotine [2].

The prevalence of EC use among adults in the United States has seen a gradual increase over the years. The use of EC has been noted to be higher among current conventional cigarettes (CC) smokers than among former or never CC smokers, and is seen to vary across states (16.2-28.4%) [3].

According to a recent report based on the Behavioral Risk Factor Surveillance System, there are about 3.62 million users of ECs among middle and high school attending students [4]. The use of EC has increased by 78% among high school students (11.7% to 20.8%) and 48% among middle school students (3.3% to 4.9%) from 2017 to 2018 [5]. This considerable increase in the youth and middle school students is attributed to the wide variety of flavors available (81% cited it as a primary reason) and also due to it being marketed either as candy or a substance safer than cigarettes [6].

However, there is growing evidence of adverse health outcomes associated with the use of ECs. The systemic effects of EC involve the respiratory, cardiovascular, gastrointestinal, nervous, and immune systems. There are studies that also demonstrate teratogenic effects, nicotine poisoning, and mechanical injury caused by EC [7].

The teratogenic effects of EC have been documented by in vitro studies that have shown embryonic cells to be more sensitive to EC refill fluids than differentiated adult cells of the lungs [8]. Negative prenatal and postnatal effects on lung growth and adult behavioral patterns in mice have also been seen with EC exposure [9, 10].

Common documented adverse effects on the GI system include xerostomia, oral mucositis, tongue discoloration, gingivitis, gum bleeding, nausea, vomiting, gastric burning, and altered bowel habits [11]. We intended to study the effects of EC use on the GI system in the US population. The study was focused on the use of ECs within the past 30 days and its effect on the GI system.

This article was previously presented as a meeting abstract at the American College of Gastroenterology, 2019 Annual Scientific Meeting & Postgraduate Course at San Antonio, Texas, on October 25, 2019, and published as an abstract in the American Journal of Gastroenterology (DOI: 10.14309/01.ajg.0000601512.93240.1d).

## Materials and methods

A retrospective review of the National Health and Nutrition Examination Survey (NHANES) data from 2017-18 was conducted. NHANES is a survey, conducted by the National Center for Health Statistics, a program under the aegis of the Centers for Disease Control and Prevention, with the primary objective of assessing the health and nutritional status of adults and children in the United States. The survey is a combination of home-based interviews and physical examinations conducted at specially designed and equipped mobile centers. The data is free of identifiers and is available in the public domain [12].

Data regarding the use of EC in the preceding 30 days were obtained from the NHANES data set and analyzed by inferential statistics using SAS 9.4 (2013; SAS Institute Inc., Cary, North Carolin, US). The data was collated through the responses obtained via a survey for assessing the smoking status of individuals using the question “During the past 30 days, on how many days did (you/SP) use e-cigarettes?”. The data for a history of vomiting and diarrhea over a period of 30 days was analyzed. The information obtained was based on the individual responses to the survey question “Did (you/SP) have a stomach or intestinal illness with vomiting or diarrhea that started during those 30 days?”, which was collected for assessing the current health status of the participants.

Additionally, data regarding age and sex were also analyzed. A p-value of <0.05 was considered statistically significant. Continuous variables were analyzed using the two-sample t-test, and categorical variables were analyzed using the Chi-Square test. All the analyses were done in SAS 9.4.

## Results

A total of 944 participants were included in the study. Of these, 261 participants used EC at least one day and 683 participants never used EC, during the preceding 30 days. Males constituted 62.84% of those using EC in our study period and 54.76% of those who never used EC in that period. Amongst EC users (n=261), 10.73% had a stomach or intestinal illness manifesting as vomiting or diarrhea that started during those 30 days compared to 8.64% who never used EC during the same period (n=683). However, the results did not reach statistical significance (p = 0.3208) (Table 1).

**Table 1:** E-cigarette use and incidence of vomiting and diarrhea in a 30-day period

	Used e-cigarettes at least one day during the past 30 days (n=261)		Never used e-cigarettes during the past 30 days (n=683)		p-Value
Had a stomach or intestinal illness with vomiting or diarrhea that started during those 30 days? (number, %)	28	10.73%	59	8.64%	0.3208
Age					
Age (<80) (mean (SD), range)	36.3 (15.0)	18 - 76	38.6 (15.1)	18 – 78	0.0324
Age >=80 (number, %)	0	0.00%	8	1.17%	n/a
Gender (male) (number, %)	164	62.84%	374	54.76%	0.0250

## Discussion

ECs, JUUL (Juul Labs, Inc., Washington D.C., United States), vape pens, and other electronic nicotine delivery systems (ENDS) have gained popularity among the population, in general, and the youth, in particular as they have been marketed by the manufacturing companies to be safer than CCs and, hence, marketed to promote their use for smoking cessation. According to a survey, they were preferred over CC, as they are perceived to be a lesser social nuisance compared to CC [13]. Other studies in the past have established a shorter in-air half-life of EC smoke in contrast to CC, thus, reducing the risks associated with passive smoking [14]. The EC users also claimed to have lesser cigarette cravings [15], fresher breath, a decreased desire to smoke, suppression of abstinence symptoms, and reduced depression, anxiety, and irritability with EC use while quitting CC [16,17]. ECs have also been recommended by physicians for pregnant women in place of CC, as a less harmful alternative [18,19].

For the longest, ECs were considered a safe alternative to CC as EC aerosols contain fewer chemicals than conventional tobacco smoke and, thus, unlike other tobacco-containing products, were unregulated. However, in 2016, the FDA successfully imposed regulations on EC use, encompassing the ingredients, health risks, product features, and use by minors. The FDA regulations also included the manufacturing, import, packaging, labeling, advertisement, promotion, sale, and distribution of ENDS, including components and parts of ENDS, but excluding its accessories [20].

However, there are conflicting views regarding safety as the short and long-term effects of EC use are poorly understood. Recent studies have shown adverse health effects of EC, which can be broadly categorized into mechanical injury, nicotine poisoning, either accidental or intentional, and systemic effects, mainly involving the respiratory, gastrointestinal, cardiovascular, neurological, and the immune system [7].

**Table 2:** Adverse effects of EC components EC: electronic cigarettes

EC Component	Clinical manifestation
Nicotine	Tachycardia, transient increase in blood pressure, dyslipidemia, coronary and systemic vasoconstriction, insulin resistance
Propylene glycol and Glycerin	Eye and respiratory irritation, pulmonary toxicity
Nitrosamines	Carcinogenic
Trace elements and heavy metals (Cadmium, Nickel, Tin, Copper, Silver, Iron, Chromium)	Pulmonary fibroblast cytotoxicity, pneumoconiosis [21], lung and sinus cancer [22]

An in vitro study by Yu et al. has shown that aerosols from EC have been implicated in DNA strand breakage and reduced cell survival [23]. In various animal studies, EC exposure has also shown a negative prenatal and postnatal effect on lung growth [9,10]. In addition, there has been documentation of aerosols generated from EC causing a decline in endothelial barrier function in cultured lung microvascular endothelial cells, leading to increased oxidative stress and inflammation in mice [9,24], with evidence of eosinophilia, increased immunoglobulin E (IgE) and inflammatory cytokine production [25]. A chemical analysis of EC has exhibited the presence of various toxic and carcinogenic components, albeit in lower concentrations [26].

We have found only limited case reports describing the effects of EC on the GI system, which paint a contrasting picture. A case report by Camus et al. shows a relapse of ulcerative colitis four months after initiation of EC use [27], while another case report by Lee et al. demonstrated clinical steroid-free remission in refractory ulcerative colitis, which began shortly after cessation of CC use [28]. Evidence of the development of necrotizing enterocolitis in an infant has also been noted with EC use by the mother during pregnancy and active labor [29]. These limited and conflicting studies highlight the need to delve further into the effects of EC use on the GI system.

Although our study could not reach statistical significance due to the limitations discussed later, this retrospective study shows a potential association between EC use and a GI pathology manifesting as either vomiting or diarrhea. Although recent studies have shown no effect of EC on the oral or gut microbiota [30], our study findings could be attributed to EC-induced alteration of motility, irritation, or possible inflammation of the GI tract.

The results of our study are to be considered taking into account its various limitations: small sample size, shorter duration of data collection, degree of EC usage in a day, presence of co-morbidities, undefined nicotine content, other conditions causing GI illness, and lack of follow-up of the study population for evaluation of long-term effects of EC exposure.

## Conclusions

In conclusion, this retrospective study shows an association between EC use and a GI pathology manifesting as either vomiting or diarrhea, which could be attributed to EC induced alteration of motility, irritation, or possible inflammation of the GI tract. However, further studies that address the limitations of our study are required to establish a causal relationship and enunciate the mechanism by which EC components affect the GI tract. Careful consideration and diligence about the health effects of ECs are required before it is assumed to be safe as a cigarette substitute or as a means of smoking cessation.
